# Use of Recombinant Antigens for the Diagnosis of Invasive Candidiasis

**DOI:** 10.1155/2008/721950

**Published:** 2008-03-11

**Authors:** Ana Laín, Natalia Elguezabal, Elena Amutio, Iñigo Fernández de Larrinoa, María Dolores Moragues, José Pontón

**Affiliations:** ^1^Departamento de Inmunología, Microbiología y Parasitología, Facultad de Medicina y Odontología, Universidad del País Vasco, Apartado 699, 48080 Bilbao, Vizcaya, Spain; ^2^Departamento de Enfermería I, Universidad del País Vasco, Barrio Sarriena s/n, 48940 Lejona, Vizcaya, Spain; ^3^Servicio de Hematología, Hospital de Cruces, Plaza de Cruces s/n, 48903 Cruces, Barakaldo, Spain; ^4^Departamento de Química Aplicada, Facultad de Química, Universidad del País Vasco, Paseo de Manuel de Lardizabal y Uribe n°3, 20018 San Sebastián, Gipuzkoa, Spain

## Abstract

Invasive candidiasis is a frequent and often fatal complication in immunocompromised and critically ill patients. Unfortunately, the diagnosis of invasive candidiasis remains difficult due to the lack of specific clinical symptoms and a definitive diagnostic method. The detection of antibodies against different *Candida * antigens may help in the diagnosis. However, the methods traditionally used for the detection of antibodies have been based on crude antigenic fungal extracts, which usually show low-reproducibility and cross-reactivity problems. The development of molecular biology techniques has allowed the production of recombinant antigens which may help to solve these problems. In this review we will discuss the usefulness of recombinant antigens in the diagnosis of invasive candidiasis.

## 1. INTRODUCTION

Invasive candidiasis is one of the leading infective complications found in immunocompromised and critically ill patients, and is associated with high morbidity and mortality. The diagnosis of disseminated candidiasis remains difficult, since signs and symptoms of the disease are unspecific and many patients with invasive *Candida* infections (up to 50%) show negative results by blood culture [1, 2], which, in addition, may only become positive late in the infection. Other standard techniques for the diagnosis of invasive candidiasis, including microscopic visualization of the infecting fungus and histopathology, usually lack specificity or sensitivity, or may require invasive procedures that can not be accomplished due to the critical conditions of many of these patients. Therefore, the diagnosis of invasive candidiasis should be based on the combined interpretation of the patient's risk factors to develop this disease, clinical manifestations (usually the presence of fever that persists despite the administration of wide spectrum antibiotics), and laboratory data (blood cultures, antibody titers).

Serological diagnosis of human infections is based on two strategies: the detection of antigens from the infecting agent in host's samples, and the detection of the antibody response elicited by these antigens in the host. This second approach is being widely studied in the diagnosis of many infectious diseases caused by a large number of microorganisms, including fungi [3, 4], bacteria [5, 6], or viruses [7].

Serological tests have been subject of much study but in many cases they can be difficult to interpret. The investigations concerning *Candida* antigen detection in serum samples show that these methods are in general quite specific but most antigens are often rapidly cleared from the circulation, so that antigen detection tests may lack the desired level of sensitivity required for a definitive diagnosis. Other serological tests sometimes require hardworking procedures that make them practically impossible to establish as routine techniques in the clinical laboratory [8, 9]. The specific antibody response that is usually induced in patients with invasive *Candida* infections can help in the diagnosis. However, antibody detection methods can also have limitations. Circulating antibodies may occur in normal individuals as a result of commensal colonization of mucosal surfaces, leading to the presence of false positive results. Thus a serological test based on the detection of anti-*Candida* antibodies should be able to distinguish between the level of antibodies detected in healthy individuals and in patients without invasive candidiasis from those detected in patients with an invasive *Candida* infection. Another problem with the clinical usefulness of antibody detection is the occurrence of false negative results in immunocompromised patients who may produce low or undetectable levels of antibodies. Anyhow, all these problems could be solved, at least in part, by the use of suitable antigens and the development of more sensitive antigen or antibody detection techniques.

Traditionally, the techniques developed to detect antibodies in patients with invasive fungal infections have made use of crude extract mixtures, composed of a large number of fungal antigens [10]. Although these extracts were easy to obtain, they did not allow standardization and facilitated cross reactivity between antibodies of patients with different invasive fungal and bacterial infections. However, over the last decades, molecular biology techniques have allowed the production of recombinant antigens. These antigens can be produced in a prokaryotic host in large amounts making standardization processes easier, and eliminating the cross-reactivity due to posttranslational modifications.

The advances in molecular biology, genomics, proteomics, and bioinformatics are helping in the design of new strategies for the development of more sensitive and specific diagnostic tests. The selection of new biomarkers for the diagnosis of systemic candidiasis has been strongly supported by the combination of proteomics and bioinformatics [11]. The recent completion of *Candida albicans* genome sequence has also been achieved thanks to advances in molecular biology and genomics [12]. All the above mentioned tools can be employed to select more specific fungal antigens and to produce them as recombinant proteins of high quality to be used for serological diagnosis.

In the present article, we review the recombinant antigens that have been investigated so far for their potential use in the diagnosis of invasive candidiasis.

## 2. RECOMBINANT ANTIGEN METHODOLOGY

When using molecular biology techniques to produce a recombinant antigen for future use in serodiagnosis, the first step is to choose the expression system and design the DNA constructs. An excellent source for the design of the DNA constructs in order to achieve the isolation of the gene of interest is the *Candida* Genome Database (CGD), where the entire sequence of the gene can be assessed by inserting its name (http://www.candidagenome.org). After having access to the sequence of the gene, knowledge of the protein to be expressed is important. Features to be considered are glycosylation states and sites, occurrence of disulphide bonds, posttranslational modifications, or overall stability of the protein.

The election of the vector and host strain is also important. Most commercial plasmids work well containing powerful promoters that show a minimal basal expression under certain conditions and that suffer a fast and dramatic induction upon a change in growth conditions of the host. Normally T7 bacteriophage promoter is used for the expression of recombinant proteins in *Escherichia coli*, being induced by IPTG. High levels of expression are normally achieved with this option. There is a large selection of vectors commercially available so it should be easy to find one with a suitable combination of markers, cloning sites, and epitopes or tags that can be used as targets for affinity purification. For this reason, when choosing the vector one should consider the size of the insert, the purification strategy to be used once the protein is expressed, the restriction sites included in the polylinker, or the antibiotic resistance for selection, among other factors.

*E. coli* is a suitable host for proteins that are not glycosylated or when glycosylation is not necessary. This is the case in some mannosylated antigens of *Candida* used in the diagnosis of invasive candidiasis, where glycosylation is not desired since cross-reactivity has been observed with antibodies that are directed to glycosylated regions of proteins, rendering them less specific. *E. coli* BL21 is a protease-deficient host strain yielding intact full-length recombinant proteins [13], and it has been used in many recombinant antigen production strategies [14–16]. Once the sequence of the gene is known and the vector is chosen, primers can be designed in order to isolate the entire gene or a specific domain of interest (e.g., the amino-terminal fragment of the antigen). These primers can be used in a polymerase chain reaction (PCR) to isolate the desired part of the gene from genomic DNA of *Candida* if there are no introns or from a cDNA library. The design of the primers should include restriction sites, matching some of the sites in the polylinker region contained in thevector. After digestion of both insert and plasmid, subsequent ligation should lead to a stable expression vector that can be cloned. Upon antibiotic selection, positive colonies containing the vector should be selected. Screening of potential PCR-amplified clones for proper insert orientation and sequence analysis of positive clones must be performed, in order to confirm that a proper reading frame has been obtained and that no errors introduced by PCR are present. Once positive clones have been selected, induction of protein expression can take place. At this point, it is important to select more than one colony since not all positive colonies are able to overexpress the recombinant protein and yield can vary from clone to clone. Recombinant protein yield and solubility are also highly dependent on the specific protein sequence, as well as on the vector, host cell, and culture conditions used. Different host growth conditions can be tested to set up the optimal conditions for the induction of protein expression.

The final step in recombinant antigen technology involves purification of the protein. This process may be simplified if affinity purification tags have been included. For high-throughput processing, His-tag has been studied for purification in soluble and insoluble conditions [17]. The incorporation of a His-tag allows for generic single-step purification using nickel-nitrilotriacetate immobilized on a resin. His-tag strategy has been employed for Hwp1 purification [15]. Hemagglutinin influenza virus (HA)-tag is also widely used for affinity purification and easy detection by immunoblotting using a anti-HA antibody. HA-tag strategy was used in Ece1 purification [3, 18].

After the recombinant, protein is purified and its value in diagnosis can be assessed by different immunological methods, such as ELISA, immunoblotting, or indirect immunofluorescence.

## 3. CANDIDA ANTIGENS AND THEIR USEFULNESS IN THE DIAGNOSIS OF INVASIVE CANDIDIASIS

### 3.1. Enzymes

Antibodies against several *Candida* enzymes have been detected in sera from patients with systemic candidiasis. Some of them are available as recombinant products. Among them, enolase is the most studied and has shown a high diagnostic value (Table 1).

#### 3.1.1. Enolase

Enolase is an immunodominant glycolytic enzyme, present in the cytoplasm and, in minor amounts, in the inner layers of the cell wall of *C. albicans* [22], either in the yeast form or in the mycelial phase of the fungus. Previous work showed that this enzyme can elicit an antibody response in the infected host [23–26]. Several authors have studied the diagnostic utility of the detection of antibodies against enolase with promising results. van Deventer et al. [25] studied 76 patients with invasive candidiasis, 46 of which were immunocompromised. The detection of anti-enolase antibodies by ELISA yielded sensitivity and specificity values of 50% and 86%, respectively, in the immunocompetent group, and 53% and 78% in the immunocompromised group, suggesting that it is possible to detect anti-enolase antibodies even in immunodepressed patients. Mitsutake et al. [24] detected the presence of antibodies against *C. albicans* enolase in 27 patients with systemic candidiasis by immunoblotting. The specificity of the test was 95% and the sensitivity 62.9%, but reached 92.5% when the number of samples tested was increased. In addition, they observed anti-enolase antibodies in infections caused by several species of the genus *Candida*, including *C. albicans*, *C. parapsilosis*, *C. tropicalis*, *C. guilliermondii,* and *C. glabrata*. However, both studies were performed with a *C. albicans* native enolase preparation, which is difficult to obtain and standardize. A recombinant enolase could therefore constitute an advance in the study of the immune response to this enzyme and its diagnostic usefulness.

A number of studies have demonstrated that recombinant enolase is useful to detect antibodies in serological tests. Sundstrom and Aliaga [27] obtained a recombinant enolase which was employed to study humoral and cellular responses in vitro [19]; and Sandini et al. [14] detected antibodies to a recombinant enolase by immunoblotting in sera from seven patients with invasive candidiasis. Laín et al. [16] have recently described the performance of a new simple diagnostic ELISA test, *Candida* Enolasa ELISA IgG test, based on the detection of antibodies against a recombinant enolase in 98 patients. Since immunocompromised individuals have an increased risk for developing invasive candidiasis, and they may produce lower antibody titers, the usefulness of this test was assayed in two different populations, 47 immunocompromised, and 51 immunocompetent patients. The results were similar in both groups of patients, with sensitivity values of 78.9% and 82.6%, and specificity values of 89.3% and 78.6%, respectively, confirming the utility of the detection of antibodies against recombinant enolase for the diagnosis of invasive candidiasis, even in immunocompromised patients. In agreement with previous studies [24], we were able to detect anti-enolase antibodies in sera from patients infected with different *Candida* species.

#### 3.1.2. Secreted aspartyl proteinases

Secreted aspartyl proteinases (Saps) are proteins secreted by *C.* *albicans* and other members of the genus. They have been described as immunodominant antigens and virulence factors associated with adherence and tissue invasion and dissemination in animal models of infection [28, 29] (Table 1). Na and Song [20] described an ELISA for the detection of antibodies against Sap1 antigen in 33 patients with invasive candidiasis, reaching a sensitivity and specificity of 69.7% and 76%, respectively. More recently, Yang et al. [21] produced a hybrid phage displaying the Sap epitope VKYTS and studied its reactivity against sera from mice and patients with systemic *C. albicans* infection, by immunoblotting and ELISA assay. The sensitivity and specificity were 77% and 88.3% in an animal model of invasive candidiasis, respectively, and 60% and 85%, respectively, in patients with invasive candidiasis.

### 3.2. Hyphal-specific candida antigens

*Candida albicans* is a fungus that can grow either as yeast or in a hyphal form. The reversible transition from yeast to mycelium or morphogenesis is thought to be a key factor for the virulence of this organism in vivo [30, 31]. Since the mycelial form is associated with the invasive phase of the fungus, the detection of antibodies directed against antigens specifically expressed in this phase may provide a way for differentiating a simple colonization from a disseminated *Candida* infection. Our group has previously reported that the detection of antibodies specifically directed to antigens expressed on the *C. albicans* germ tube surface by indirect immunofluorescence has a good diagnostic value, with a sensitivity of 79–89% and a specificity of 91–100%, on both competent and immunocompromised patients [32–36]. However, this method requires the adsorption of the sera with heat-killed *C. albicans* yeast, in order to eliminate the reactivity with the antibodies directed to the yeast form [37]. These antimannan antibodies are commonly found in humans and are responsible for the majority of the false positive results observed in many serological studies. The production of hypha-specific recombinant antigens in a prokaryotic host would eliminate the need to adsorbe the sera to remove the antimannan antibodies, since the recombinant protein will be nonglycosylated. A number of antigens specifically expressed on the *C. albicans* germ tube have been recently identified. We hereby describe the potential of some of them for *Candida* serodiagnosis.

#### 3.2.1. Hyr1 protein

Hyphally regulated protein 1, Hyr1, [38] is a *C. albicans* germ tube specific cell wall glycoprotein of 937 aminoacids, with a glycosylphosphatidylinositol (GPI) motive. Hyr1 is specifically expressed in the mycelial form and its heterologous expression in *Saccharomyces cerevisiae* did not show an obvious phenotype. Consequently, it was suggested to have a structural role in the *C. albicans* cell wall architecture.

Laín et al. [18, 39] studied the reactivity of a recombinant Hyr1 protein with sera from 36 patients with invasive candidiasis and 45 control patients by immnunoblotting. The sensitivity and specificity values of the assay were 39.1% and 100%, respectively, (Table 2). The detection of anti-Hyr1 antibodies by an ELISA assay increased the sensitivity to 58.3%, but reduced the specificity to 82.2% in the diagnosis of invasive candidiasis (Table 2).

#### 3.2.2. Ece1 protein

Extent of cell ellongation 1, Ece1 [40], is another *C. albicans* germ tube specific protein. An *ECE1* null mutant *C. albicans* strain did not show morphological changes and the authors concluded that this protein is not essential for cell elongation or hyphal formation, despite the strict association of Ece1 expression with the mycelial form of *C. albicans*.

By using a similar approach to that followed to study the diagnostic potential of the Hyr1, the *ECE1* gene has been expressed in *E. coli* and used in both immunoblotting and ELISA assays to detect antibodies in patients with invasive candidiasis [18, 41]. As observed with the Hyr1 antigen, the results obtained by ELISA showed higher sensitivity but lower specificity than those obtained by immunoblotting in the diagnosis of invasive candidiasis (Table 2).

#### 3.2.3. Als3 protein

The *ALS* (agglutinin-like sequence) gene family of *Candida albicans* encodes cell surface glycoproteins implicated in adhesion of the organism to host surfaces. *ALS* genes conform a basic three-domain structure that includes a relatively well conserved domain of 1299 to 1308 nucleotides (433 to 436 aminoacids), a central domain of variable length consisting entirely of a tandemly repeated 108-bp motif, and a C-terminal domain of variable length and sequence that encodes a serine-threonine rich protein, with a GPI motif. Presently, eight genes in the *ALS* family have been reported in the literature [42]. *ALS* genes are differentially regulated by physiological conditions such as changes in the growth medium, morphogenesis, or the growing phase of the fungus. The expression of these proteins is correlated with *Candida* infection, and there has also been found evidence of these proteins in other *Candida* species, such as *C. dubliniensis* and *C. tropicalis* [43]. Among the Als family, *Als*3 is implicated in endothelial and epithelial adhesion and it is specifically expressed in the *C.* *albicans* germ tube [44]. Several authors have established a functional model for the *Als* proteins, where the N-terminal domain is exposed to the cell surface, and the central and C-terminal domain are integrated in the cell wall layers [42, 45]. The N-terminal domain of the *Als*3 that has been described as a binding motif [44] is the most exposed part of the protein and therefore to the immune system of the host. However, the detection of antibodies against the N-terminal recombinant fragment of the *Als*3 for the diagnosis of invasive candidiasis has shown a very poor diagnostic value (Table 2) [18].

#### 3.2.4. Hwp1 protein

Hyphal wall protein 1 (Hwp1), described by Staab et al. [46], is a glycoprotein specifically expressed in the cell wall surface of the hyphae of *C. albicans*, which has been studied as an important adhesin, required as a virulence factor in invasive candidiasis [47]. Naglik et al. [48] analysed by RT-PCR the presence of *HWP1* mRNA in human subjects who were positive for *C. albicans* culture and had oral or vaginal symptoms, as well as in asymptomatic patients, by RT-PCR. They also detected antibodies against a recombinant fragment of the Hwp1, Hwp1N13, consisting of the transglutaminase substrate domain, previously produced by Staab et al. [49]. *HWP1* mRNA was found to be correlated with the presence of *C. albicans* in both asymptomatic carriers and in cases of oral and vaginal candidiasis, although the quantity of mRNA in candidiasis probably exceeded that revealed in asymptomatic conditions. Antibody titers (IgG and IgA) in oral-culture-negative carriers and candidiasis cases were found to be equivalent, suggesting that Hwp1 is a common target of host responses to *C. albicans* that are recognized to result from long-term colonization.

Since this protein has a surface-exposed N-terminal domain that binds antibodies, while the carboxyl terminus is most probably covalently integrated in the cell wall [46], Laín et al. [15] selected the N-terminal fragment of the Hwp1 (161 aminoacids) for serological studies in patients with invasive candidiasis. While detection of antibodies by immunoblotting showed very poor sensitivity and negative predictive values (27.8% and 62.3%, resp.), detection of antibodies against the N-terminal fragment of Hwp1 by ELISA notably increased the diagnostic usefulness, with sensitivity, specificity, positive and negative predictive values of 88.9%, 82.6%, 80.0%, and 90.2%, respectively. Interestingly, these results were very similar to those obtained by the detection of antibodies to the *C. albicans* germ tube by indirect immunofluorescence, a technique that has been previously reported to be of diagnostic utility [35, 50]. However, the detection of antibodies against the Hwp1 recombinant fragment is easier to perform, since it does not require the adsorption of the patient's serum and allows both an objective measurement and automatization of the test. In addition to that, the ELISA test was able to detect invasive candidiasis in patients infected by non-*C. albicans* species, including *C. parapsilosis*, *C. tropicalis*, *C. utilis*, *C. glabrata,* and *C. dubliniensis*, although this antigen was initially described as specific of the germ tube of *C. albicans*. The reasons for this reactivity are not known at present, but it has been reported that *HWP1* mRNA may also arise from pseudohyphal and yeast growth forms in *C. albicans* [48], and an ORF of 1266 pb with homology to *C. albicans* Hwp1 has been found in *C. dubliniensis* [51]. We have also observed that the region encoding the first 85 residues of the Hwp1 fragment shows homology with the sequence of proteins of non *albicans Candida* species [15].

## 4. CONCLUSIONS AND FUTURE PERSPECTIVES

**Figure 1:**
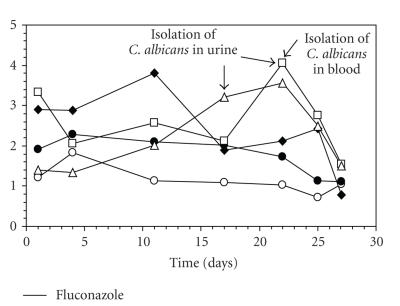
Kinetics of antibody levels against different *C. albicans* recombinant proteins in a patient with invasive candidiasis. Ece 1 (*O*), Hyr1 (♦), Als3 (•), enolase (□), and Hwp1 (Δ). Arrows point to the days where cultures of urine and blood yielded *C. albicans*. The bar shows the days the patient was treated with fluconazole.

There is an increasing interest in the development of new, reliable, and simple diagnostic tests for the diagnosis of invasive candidiasis. Molecular biology techniques have allowed the production of recombinant antigens which may be useful for the detection of antibodies against them. It has been demonstrated that the detection of antibodies against purified and well-defined recombinant antigens allows the diagnosis of invasive candidiasis. A mixture of different antigens in the same assay may optimize and increase the diagnostic value of these tests, since the kinetics of antibodies against each recombinant antigen in the same patient is different (Figure 1). Identification of the most immunogenic domains of each antigen can also improve the results. A combined detection of antigen and antibody may also increase the sensitivity. Finally, further search and production of new immunogenic recombinant antigens could open new ways for an accurate diagnosis of invasive candidiasis.

## Figures and Tables

**Table 1:** *Candida* recombinant antigens tested for serodiagnosis of invasive candidiasis.

Antigen	Molecular mass (kDa)	Reference
Enzymes		

Enolase	48	[14, 16, 19]
Sap	—	[20, 21]

Hypha specific antigens		

Ece1	35	[18]
Hyr1	90	[18]
N-Als3	50	[18]
Hwp1	61	[15]

**Table 2:** Diagnostic usefulness of the detection of antibodies against Hyr1, Ece1, Als3, and Hwp1 by immunoblotting and ELISA assay [18] (PPV: positive predictive value, NPV: negative predictive value).

	Sensitivity%	Specificity%	PPV%	NPV%
Immunoblotting				

Hyr1	39.1	100	100	76.3
Ece1	34.8	93.3	72.7	73.7
Als3	26.1	93.3	66.7	71.2
Hwp1	27.8	97.5	83.3	62.3

ELISA assay				

Hyr1	58.3	82.2	72.4	71.2
Ece1	55.6	80.0	69.0	62.5
Als3	41.7	77.8	60.0	62.5
Hwp1	88.9	82.6	80.0	90.2
